# Cytokine-Induced Senescence in the Tumor Microenvironment and Its Effects on Anti-Tumor Immune Responses

**DOI:** 10.3390/cancers14061364

**Published:** 2022-03-08

**Authors:** Maximilian Rentschler, Heidi Braumüller, Priscilla S. Briquez, Thomas Wieder

**Affiliations:** 1 Department of Dermatology, University Medical Center Tübingen, Eberhard Karls University Tübingen, 72076 Tübingen, Germany; maximilian.rentschler@med.uni-tuebingen.de; 2 Department of General and Visceral Surgery, Medical Center—University of Freiburg, Faculty of Medicine, University of Freiburg, 79106 Freiburg, Germany; heidi.braumueller@uniklinik-freiburg.de (H.B.); priscilla.briquez@uniklinik-freiburg.de (P.S.B.); 3 Department of Vegetative and Clinical Physiology, Institute of Physiology, Eberhard Karls University Tübingen, 72074 Tübingen, Germany

**Keywords:** cell cycle regulation, cell death, growth arrest, chemoresistance, immunotherapy, T cells, inflammatory cytokines, senescence surveillance, tumor dormancy, tumor microenvironment

## Abstract

**Simple Summary:**

Despite tremendous treatment efforts, cancer is still one of the leading causes of death, with approximately 10 million deaths in 2020. In the last decade, immunotherapy entered the stage of clinical practice and was added to the established regimen, i.e., surgery, chemo- and radiation therapy, to fight this deadly disease. Cancer immunotherapies, including immune checkpoint inhibitors, target malignant cancer cells and immune cells in the tumor micro-environment. Among those cells are T cells and antigen-presenting cells, which can efficiently control tumors via both cell-cell interactions and by secretion of inflammatory cytokines. The presence of specific cytokines in the tumor microenvironment has been shown to induce senescence in tumor cells. Subsequently, tumor cells acquire a senescence-associated secretory phenotype that strongly modulates anti-tumor responses. This review describes the mechanisms of cytokine-induced senescence in the tumor microenvironment and highlights their relevance for therapeutic perspectives.

**Abstract:**

In contrast to surgical excision, chemotherapy or radiation therapy, immune checkpoint blockade therapies primarily influence cells in the tumor microenvironment, especially the tumor-associated lymphocytes and antigen-presenting cells. Besides complete remission of tumor lesions, in some patients, early tumor regression is followed by a consolidation phase where residing tumors remain dormant. Whereas the cytotoxic mechanisms of the regression phase (i.e., apoptosis, necrosis, necroptosis, and immune cell-mediated cell death) have been extensively described, the mechanisms underlying the dormant state are still a matter of debate. Here, we propose immune-mediated induction of senescence in cancers as one important player. Senescence can be achieved by tumor-associated antigen-specific T helper 1 cells, cytokines or antibodies targeting immune checkpoints. This concept differs from cytotoxic treatment, which often targets the genetic makeup of cancer cells. The immune system’s ability to establish “defensive walls” around tumors also places the tumor microenvironment into the fight against cancer. Those “defensive walls” isolate the tumor cells instead of increasing the selective pressure. They also keep the tumor cells in a non-proliferating state, thereby correcting the derailed tissue homeostasis. In conclusion, strengthening the senescence surveillance of tumors by the immune cells of the microenvironment is a future goal to dampen this life-threatening disease.

## 1. Introduction

**Figure 1:**
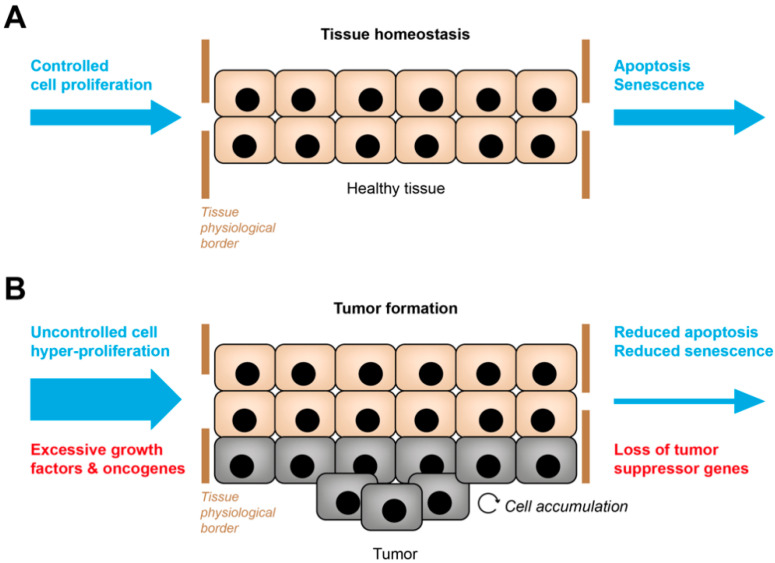
Deranged tissue homeostasis in hyperplastic tumors. (**A**) Under physiological conditions, the size of a specialized tissue is kept constant (beige cells). Tissue homeostasis is a steady state where tissue generation by cell proliferation (blue arrow on the left) and clearance of damaged or old cells by apoptosis or cellular senescence (blue arrow on the right) are kept in balance. (**B**) High levels of growth factors or the activation of oncogenes lead to hyperproliferation (enlarged blue arrow on the left), and loss of tumor suppressors cause reduced apoptosis or cellular senescence (narrowed blue arrow on the right). This dysfunctional tissue homeostasis evokes excessive tissue formation and hyperplastic tumor growth (grey cells).

Tissue homeostasis is a dynamic process regulated by a delicate balance between tissue generation, characterized by cell proliferation, differentiation, and clearance mechanisms of damaged, old or dead cells, mainly those that underwent apoptosis or cellular senescence (Figure 1A) [1]. While apoptosis is a form of programmed cell death, cellular senescence is a terminal state where the cell becomes resistant to apoptosis. Thus, it remains viable but is permanently growth-arrested and unable to re-enter the cell cycle despite stimulation by growth factors [2,3,4,5,6]. Nevertheless, it has also been shown that growth-arrested cancer cells can escape from senescence and give rise to even more aggressive tumors [7,8,9,10]. Another hallmark of senescence is that the cells remain metabolically active and secrete multiple pro-inflammatory and pro-regenerative factors, including chemokines, cytokines, growth factors and proteases, thereby adopting a so-called senescence-associated secretory phenotype (SASP) [11,12]. The SASP induces substantial changes in the tissue microenvironment, notably by attracting immune cells to clear the senescent cells and by promoting tissue regeneration to replace them (e.g., by modulating stem and progenitor cells, by inducing angiogenesis or by rearranging the extracellular matrix (ECM)). Together, the SASP participates in the proper maintenance of tissue homeostasis [13]. Besides these beneficial traits, there is also a “dark side” of the SASP, as it can exert deleterious effects leading to tumor promotion or mediating senescence escape [11,14,15]. In adults, cellular senescence generally occurs as a response to intrinsic or extrinsic cellular stresses, such as DNA damage, dysregulated mitogenic signaling, telomere shortening, epigenetic changes, inflammatory signals, or exposure to ionizing radiation or genotoxic substances. In addition, experimental evidence suggests that cellular senescence is an evolving process leading to a diversity of senescent cell phenotypes [16]. Importantly, the different triggers and multiple stages of cellular senescence, the specific cell type, and the cell-to-cell variability are well-known factors that modulate the composition of the SASP, thereby affecting the efficacy of immune-mediated clearance and tissue homeostasis [17].

Dysregulation of tissue homeostasis can lead to excessive cellular tissue formation, for example, in the case of tumor development. Although it has been long known that tumorigenesis is a complex multi-step process rather than simple tissue overgrowth, it used to be the activation of oncogenes and the repression of tumor suppressor genes that best described the molecular events underlying tumor formation [18,19] (Figure 1B). In 2000, a groundbreaking review by Hanahan et al. highlighted the complexity of malignant tumor cells (i.e., cancer cells) by describing six cancer hallmarks [20]: the cancer cells display growth factor-independent proliferation, evade growth suppression, resist cell death, show signs of replicative immortality, induce angiogenesis, and activate cellular programs that enable themselves to invade other tissues and metastasize to distant organs. All these hallmarks were mainly coined from the perspective of the malignant tumor cells. Later, the authors added multiple hallmarks of cancer, such as tumor evasion from immune surveillance [21], this time underscoring the central role of the tumor microenvironment (TME) during cancer progression. Indeed, tumors contain not only cancer cells but also a repertoire of “normal” non-cancerous cells, specifically immune and stromal cells [22,23]. Interestingly, many types of cancer cell-associated damage, such as genomic instability and mitogenic dysregulations, are potential triggers of cellular senescence. In fact, senescent cells are present in most, if not all, cancers and have been very recently added to the list of cancer hallmarks [24]. Senescence is commonly considered a protective tumor-suppressive mechanism, as it prevents the proliferation of damaged cancer cells and the progression from pre-malignant to malignant tumors. In addition to intrinsic cellular damage, cellular senescence can be induced by immune cells upon the secretion of particular cytokines. Cytokine-induced senescence (CIS) by immune cells has been established as an important barrier to stop malignant tumor growth [25,26]. Nevertheless, cellular senescence also leads to the secretion of the SASP, known to be pro-tumorigenic, as it regulates anti-tumor immune responses and enhances tumor angiogenesis and growth [11,27]. Since senescence can also occur in non-cancerous cell types of the TME (i.e., stromal and immune cells), this adds another layer of complexity to decipher the role and the effects of cellular senescence in cancer.

In this review, we detail how CIS affects tumor and non-cancerous cells in the TME and how their SASP modulates the TME. We particularly discuss the important role of CIS and its SASP for anti-tumor immune responses and highlight the necessity of clearance of senescent cells to optimize the long-term efficacy of cancer therapies.

## 2. The Tumor Microenvironment (TME)

Solid tumors are made of a complex and dynamic environment wherein tumor cells interact with immune cells, stromal cells and the extracellular milieu, including the ECM and cell-secreted soluble biomolecules. In addition, many tumors have been shown to contain a microbiome, such as colorectal cancer, skin cancer, and breast cancer, to cite a few [28], adding to the complexity of the TME. Although cellular senescence has been studied in detail in tumor cells, it can directly or indirectly affect the behavior of many different cell types. In this section, we provide an overview of the TME to set the stage for further understanding of tumor dormancy. More comprehensive reviews focusing on the composition and complexity of the TME in a broader context can be found in [29,30].

### 2.1. Tumor Cells

Despite being from the same cancer type, tumor cells are highly heterogenous between patients, between different tumors of the same patient and even within a single tumor. This heterogeneity is associated with their high rate of genetic mutations and epigenetic modifications, their increased proliferation rate as well as other interactions within the surrounding microenvironment. Importantly, the presence of cancer stem cells in tumors, which share some characteristics of normal stem cells, has been shown to induce multi-lineage cell populations, which commonly promote tumor growth, tumor cell heterogeneity, and teratoma formation [31,32]. Besides, cancer cells can also be found in a living yet dormant state, either being quiescent or senescent. Quiescence is reversible, with the quiescent cells being able to re-enter the cell cycle in a relatively short time upon stimulation with specific signals (e.g., growth factors) depending on the depth of the quiescence [33]. In contrast, senescence is generally regarded as an irreversible process that is characterized by permanent cell growth arrest, although escape from senescence has been described. Nevertheless, senescent cells remain active in the secretion of bioactive signals (e.g., cytokines, proteases, growth factors) and can adapt stem cell functions [7]. Tumor cells commonly gather tremendous differences in their cellular phenotypes. It is, therefore, necessary to keep in mind that the high plasticity and heterogeneity of tumor cells allow for tumor escape upon therapy, leading to incomplete response and cancer relapse. This remains one of the biggest challenges to overcome in future cancer treatment regimens. Indeed, inducing selective pressure on some tumor cell subpopulations can favor both their phenotypic changes and the development of other tumor cell subsets, leading to a tumor with different characteristics than the initial one [34]. Similarly, while primary and metastatic tumors share a common origin, the important behavioral changes required for a cancer cell to escape a primary tumor and form a metastasis (e.g., extravasation, circulation, and invasion) can lead to substantial differences in the primary vs. metastatic tumor cell populations. In this case, the tissue or organ targeted by the metastatic cells additionally shape the tumor development, modulating the immune cells, stromal cells and ECM composition and activities in the metastatic tumor [35,36].

### 2.2. Immune Cells in the TME

The immune cell composition of the TME includes tumor-infiltrating lymphocytes (TILs), tumor-associated macrophages (TAMs), tumor-associated neutrophils (TANs), and myeloid-derived suppressor cells (MDSCs). With the induction of novel therapies such as immunotherapies, the focus of anti-tumoral treatments changed from targeting tumor cells directly to targeting cells of the TME. This paradigm shift brought the specialized immune cells of the TME into focus, as immunotherapeutics indirectly kill tumors via immune cells and non-immune cells. Thus, analyzing the cell types and molecules present in the tumor will shed light on the mechanisms of successful tumor control.

#### 2.2.1. Tumor-Infiltrating Lymphocytes (TILs)

TILs consist of several subsets of immune cells, including T cells, innate lymphoid cells like natural killer cells (NKs) and B cells. All these cells circulate in the bloodstream and have to migrate into the tumor, where they can exert both pro- and anti-tumorigenic functions. The composition of TILs is very variable depending on the tumor type.

*Natural killer cells (NKs).* NKs are innate lymphoid cells with cytotoxic functions similar to CD8^+^ T cells but without clonotypic receptors. NKs kill their targets by releasing granzymes and perforin without antigen-specific activation [37]. To prevent the killing of healthy cells, NKs possess inhibitory receptors and NK-activating receptors [38]. Inhibitory receptors interact with major histocompatibility complex class I molecules (MHC-I) and avoid killing MHC-I-positive cells. Activating receptors recognize molecules derived from pathogens, viruses or growth factors [39,40]. In lung cancer and gastrointestinal cancer, NKs have altered expression of inhibitory and activating receptors and overexpress exhaustion markers like T cell immunoreceptor with Ig and ITIM domains (TIGIT) and T cell immunoglobulin and mucin domain-containing protein 3 (TIM-3). The classical and best-analyzed exhaustion marker of T cells, programmed cell death 1 (PD-1), does not seem to play a role in NK exhaustion [41,42].

*T cells.* CD8^+^ T cells recognize antigen presented on MHC-I complex upon engagement of the T cell receptor (TCR). Activation of CD8^+^ T cells takes place in the tumor-draining lymph node. There, conventional dendritic cells (cDCs) cross-present tumor antigens on MHC-I. CD4^+^ T helper cells promote further clonal expansion and differentiation of CD8^+^ T cells into effector and memory T cells [43]. CD8^+^ effector T cells can kill transformed cells by releasing perforin and granzymes or by inducing apoptosis through FAS-FAS ligand binding [44]. Depending on the cytokines they produce, cytotoxic T lymphocytes (CTLs) can be classified into Tc1, Tc2, Tc9, Tc17, and Tc22 subtypes. In solid tumors, interferon-gamma (IFN-γ)-producing Tc1 cells are the most frequently observed subtype, but different tumor types harbor different CTL subtypes [45]. In many tumors, like colorectal cancer, breast cancer, ovarian cancer or bladder cancer, the infiltration of CD8^+^ CTLs is thought to be a good prognostic marker [46]. However, in large, progressed tumors, CTLs are often exhausted and dysfunctional and express high levels of exhaustion markers like PD-1, lymphocyte-activation gene 3 (LAG-3), cytotoxic T lymphocyte-associated antigen 4 (CTLA-4) or TIGIT [47].

CD4^+^ T helper (Th) cells recognize antigens in connection with the MHC-II complex present mainly on antigen-presenting cells (APCs). As most tumor cells do not express MHC-II, CD4^+^ Th cells cannot sense tumor antigens or tumor cells directly. Nevertheless, Th cells are necessary for any anti-tumorigenic immune response as they regulate the immune functions of most of the TILs [48]. Despite these indirect functions, CD4^+^ T cells can eliminate tumor cells directly through the release of granzyme B and perforin and through the induction of apoptosis via FAS-FAS ligand [49]. Naïve CD4^+^ T cells can differentiate into several subtypes, depending on the cytokine milieu produced by APCs. In tumors, Th1 cells are most abundant, but other subtypes like Th2, Th17, Th9, Th22 and regulatory T cells (Tregs) are also present [50]. IFN-γ-producing Th1 cells are associated with a good prognosis in several cancer types, including breast cancer, gastric cancer, hepatocellular carcinoma or lung cancer. Interleukin (IL)-4-producing Th2 cells and IL-17-producing Th17 cells are mostly associated with tumor progression in several tumor entities [46]. Both subtypes contribute to chronic inflammation, one of the factors described as a key catalyst for tumor formation and tumor progression. Enhanced numbers of Th22 cells, which produce IL-22, are associated with tumor progression through the immuno-suppressive functions of this cytokine [46].

CD4^+^ Tregs have immunosuppressive functions and are therefore tumor-promoting. They express high levels of surface CD25 and CTLA-4 and the transcription factor forkhead box protein 3 (FOXP3). Tregs suppress the immune system in two ways. First, they can exert inhibition by cell-cell interactions. The inhibitory molecule CTLA-4 on the cell surface of Tregs binds to co-stimulatory molecules on DCs. This leads to reduced T cell activation and proliferation [51]. Second, Tregs produce several immunosuppressive cytokines like transforming growth factor-beta (TGF-β) and IL-10. There are some more mechanisms to how Tregs suppress the function of immune cells in the TME, like competition with CTLs for IL-2 [52]. In most cancer types, the infiltration of Tregs is correlated with a poor prognosis [46].

*B cells.* Compared with T cells, B cells are present in the TME only in low numbers. Some studies identified a tumor-promoting role, whereas other studies showed an association with improved cancer outcomes, particularly when lymphoid organs known as tertiary lymphoid structures are formed [53,54,55,56,57]. In these tertiary lymphoid structures, B cells present tumor antigens to T cells, produce anti-tumoric antibodies, and secrete cytokines that enhance CTL functions. However, B cells can also have regulatory functions, thereby promoting tumor progression through the cytokines IL-10 and TGF- β [58].

#### 2.2.2. Myeloid Cells

*Tumor-associated macrophages (TAMs).* Tumor cells recruit circulating monocytes from the peripheral blood into tumors mainly through the secretion of colony-stimulating factor-1 (CSF-1) and monocyte chemoattractant protein-1 (MCP1; also known as C-C motif chemokine ligand 2 (CCL2)) [59]. In the TME, infiltrating monocytes get polarized into M1 and M2 macrophages. Conventionally, reactive oxygen species (ROS)-producing M1 macrophages are regarded as inhibitors of tumor growth, whereas IL-10 and TGF-β producing M2 macrophages are thought to promote tumor growth. In most tumors, the presence of M2 macrophages correlates with a poor prognosis. It is now clear that the classification of TAMs into M1 and M2 macrophages is too crude, as TAMs exist in several subtypes with very high plasticity [47].

*Dendritic cells (DCs).* DCs arise from progenitors in the bone marrow, where they differentiate into plasmacytoid DCs and immature cDCs. Immature cDCs leave the bone marrow and migrate to distant tissues, where they engulf antigens. To effectively process these antigens and present them on the MHC-II complex and via cross-presentation on the MHC-I complex, cDCs have to mature. Maturation starts upon recognition of danger-associated molecular patterns (DAMPs) or pathogen-associated molecular patterns (PAMPs). Mature cDCs express the chemokine receptor CCR7, and this directs the DC into lymphoid organs and enhances the expression of co-stimulatory molecules and MHC molecules dramatically [60]. In the T cell zone of the lymphoid organs, cDCs prime naïve T cells. There are two cDC subtypes, cDC1 and cDC2, both of which have different functions [61]. cDC1s play an important role in anti-tumor immunity both as lymph node resident cells and migratory cDCs that deliver antigens from the tumor to the lymph node. However, some intratumoral cDC1s never leave the tumor area but secrete chemokines that attract naïve and activated T cells. cDC2s present antigens on MHC-II much more effectively than cDC1s, making them better inducers of CD4^+^ T cell responses than cDC1s [62].

*Myeloid-derived suppressor cells (MDSCs).* MDSCs derive from common myeloid progenitors in the bone marrow. Myeloid progenitors give rise to the granulocyte-monocyte progenitors and myeloid-dendritic cell progenitors. The further differentiation of the progenitors into neutrophils, monocytes, and DCs is driven by specific transcription factors and growth factors [63]. However, when the differentiation is blocked by soluble factors released from the circulation, immature myeloid precursors result [64,65]. These immature precursors exhibit strong immune suppressive capacities and are called MDSCs. As MDSCs are immature, they share features of granulocytes and monocytes. In the TME, MDSCs get activated by various factors, such as vascular endothelial growth factor (VEGF), granulocyte-macrophage colony-stimulating factor (GM-CSF), matrix metallopeptidase 9 (MMP-9), IFN-γ, TGF-β, IL-1β, IL-6, IL-10, IL-12, IL-13, CCL2, C-X-C motif chemokine ligand (CXCL) 5, CXCL12 and prostaglandins [47]. Most of these factors are also members of the SASP, the secretory program of senescent cells.

*Tumor-associated neutrophils (TANs).* TANs are short-lived myeloid immune cells that originate from precursors in the bone marrow [66]. The secretion of C-X-C chemokines recruits neutrophils into tissues [67,68], but oxysterols and the complement component anaphylatoxin C5a secreted by tumor cells contribute to the recruitment of neutrophils from the blood into tumors [69,70]. In tumors, cancer cells can prolong the survival of TANs dramatically by secreting IL-1β and granulocyte colony-stimulating factor (G-CSF) [66]. G-CSF, in combination with TGF-β in the TME, induces the expression of arginase 1 (ARG1), nitric oxide (NO), and ROS. These neutrophil-derived factors inhibit the activation of T cells in the TME efficiently [67,71]. Together with the synthesis of prostaglandin E_2_ (PGE_2_) and the expression of programmed cell death ligand 1 (PD-L1), TANs are regarded to be important drivers of immunosuppression [72,73,74]. But, like other immune cells in the TME, TANs can also display anti-tumor activities. TANs can kill tumor cells directly by inducing apoptosis or by inducing lethal calcium influx [72,75,76].

### 2.3. Stromal Cells in the TME

In addition to immune cells, the tumor cells interact with stromal cells, which remodel the TME and promote tumor growth. Stromal cell populations importantly include endothelial cells, pericytes, fibroblasts and other cells, such as mesenchymal stem cells or adipocytes.

Endothelial cells are the cells lining the inner wall of blood or lymphatic vessels. In tumors, blood endothelial cells (BECs) detect the hypoxic environment induced by the high metabolism of tumor cells to create new blood vessels, a process called tumor angiogenesis. This permits increased perfusion of oxygen and nutrients in the tumor while also providing routes for cell infiltration or dissemination [77]. Similarly, lymphatic endothelial cells (LECs) undergo lymphangiogenesis to develop new lymphatic vessels. This modulates tumor immunity by increasing the draining of tumor-derived molecules and cell trafficking between the tumor and lymph nodes. Moreover, lymphatic vessels provide additional routes for tumor metastasis. While tumor blood and lymphatic vessels are functional in the tumor, their structure differs from physiologically healthy vessels; for example, the blood vasculature in the tumor is highly permeable and rather disorganized, with a partial loss of supporting basement membranes [78]. Pericytes around the tumor blood capillaries are in loose contact with the endothelial cells, and their number is dysregulated compared with healthy vessels [79]. Interestingly, pericytes not only have a role in maintaining blood vessels’ integrity but also are multipotent cells, serving as a source of stem cells in tumors [80]. They also display tumor proliferative and immunomodulatory effects via the secretion of chemokines and cytokines [81].

Another important cell type of the tumor microenvironment is cancer-associated fibroblasts (CAFs) [82]. CAFs are fibroblasts that are reactivated in the tumor, thereby orchestrating tumor development. Indeed, CAFs influence the growth of tumor cells and of blood capillaries via the secretion of growth factors. They have immunomodulatory functions via the secretion of chemokines and cytokines, and they produce and regulate the turnover of the ECM, being mostly responsible for the desmoplastic response. In addition to paracrine interactions through soluble signaling factors, CAFs have been shown to directly interact with tumor cells, immune cells or other stromal cells [83,84], adding to the complexity of their functions. Importantly, CAFs demonstrate very high cell plasticity, are multipotent, and can adopt a multitude of different phenotypes, being tumor-promoting or -restraining depending on the context [82]. As such, CAFs constitute a highly heterogeneous cell population in the tumor and remain one of the most studied stromal cell types in cancer. In addition, many other cell types can be found in the tumor stroma, such as mesenchymal stem cells (MSCs) or adipocytes.

### 2.4. The Extracellular Matrix (ECM)

The structure and composition of the ECM regulate its biomechanical and biochemical properties, which directly modulate cell behavior by providing adhesion ligands and bioactive signaling molecules, such as cytokines and growth factors [85]. The interstitial matrix is primarily made of collagen fibers intermingled with elastin fibers, a core scaffold further decorated with glycoproteins, proteoglycans, and glycosamino-glycans (GAGs). In the TME, the interstitial ECM is dysregulated and frequently acquires a desmoplastic phenotype, with increased collagen content and fiber alignment, along with an imbalance of other ECM proteins and GAGs [86]. Similarly, basement membranes are also impaired in tumors. Basement membranes are the specialized matrices underlying epithelial cells or surrounding blood vessels (and partially lymphatic vessels) and are primarily made of collagen IV and laminin [87]. In the TME, the basement membranes of blood vessels have been observed to have a partial or complete loss of integrity, particularly at the invasive front of the tumor, which strongly affects mechanical properties and alters cell intra- and extravasation [87,88]. Interestingly, the link between ECM dysregulation and cellular senescence has just started to be explored in cancer and diseases [89,90,91].

**Figure 2:**
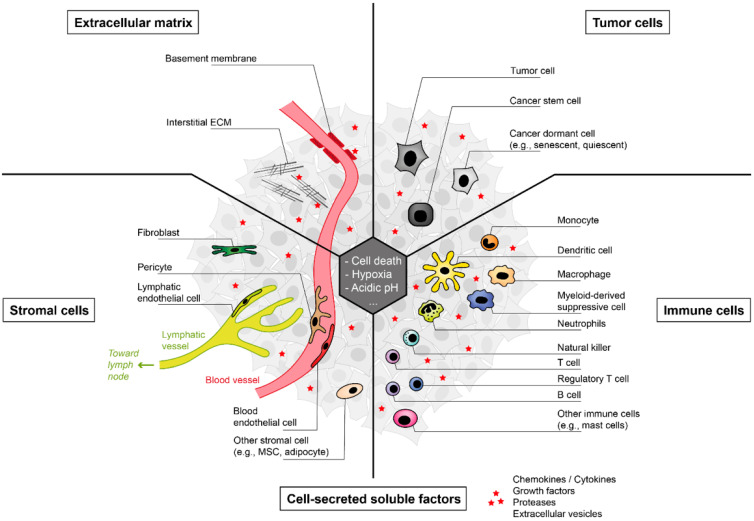
Overview of the components of the tumor microenvironment (TME). The cellular composition of the TME is quite heterogeneous and consists of various cell types. These include (i) tumor cells (upper right) as well as (ii) stromal cells (lower left) and (iii) immune cells (lower right). Soluble factors secreted by the cells of the TME (red stars) also play an important role, as do structural components, such as (iv) the extracellular matrix (ECM; upper left).

As outlined before, the TME and its composition is a quite complex network (for an overview, see Figure 2). This setting becomes even more complex regarding the molecular interactions between the different cell types found within the TME. In particular, the secretion of soluble factors by cells of the immune system or the tumor cells has a great impact on the surrounding tissue.

## 3. Immune Surveillance of Tumors by Toxic and Non-Toxic Mechanisms

**Figure 3:**
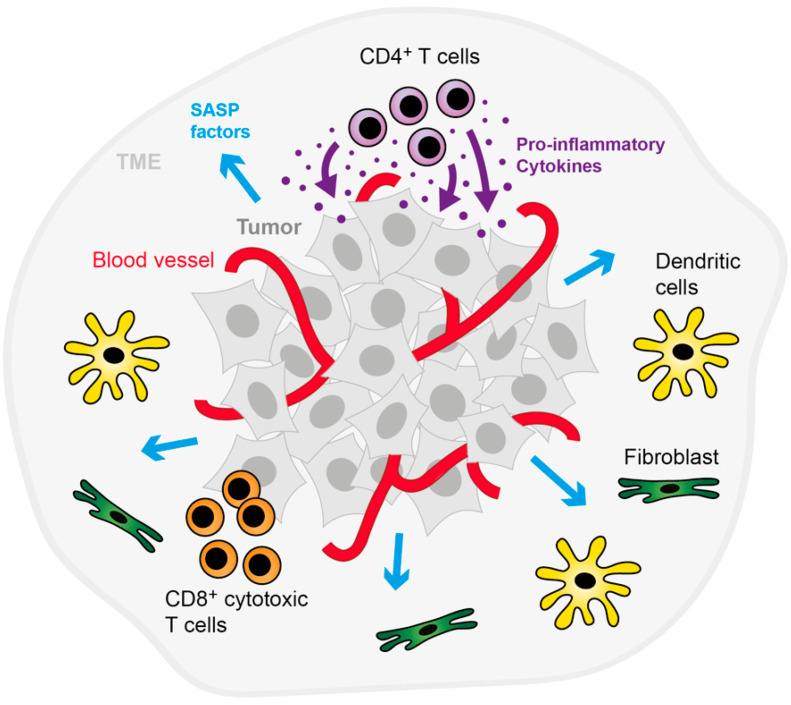
Cytotoxic and non-cytotoxic tumor immune control. The cartoon summarizes the control of a tumor by non-toxic mechanisms executed by CD4^+^ T cells that secrete pro-inflammatory cytokines (upper part of the cartoon) or toxic mechanisms executed by tumor-infiltrating cytotoxic CD8^+^ T cells (lower part of the cartoon). Abbreviations: SASP, senescence-associated secretory phenotype; TME, tumor microenvironment.

According to the “magic bullet” concept originally introduced by Paul Ehrlich more than 100 years ago, tumors should be eradicated either by excision or cellular destruction. This strategy of tumor cell killing is the basic principle of the “war on cancer” (for reviews see [92,93]) and relies on (i) induction of programmed cell death (e.g., apoptosis or necroptosis), (ii) necrotic cell death, (iii) autophagic cell death, (iv) target cell lysis, or (v) oxidative burst. This concept translated into the four current pillars of tumor therapy, i.e., complete surgical excision before the tumor has started to metastasize, eradication of the remaining tumor mass by radiation therapy, killing of disseminated tumor cells by chemotherapy, and destroying tumor cells by cytotoxic immunotherapy. With the introduction of immune checkpoint inhibitor therapy, the control of disseminated tumors by immune cell (re)activation was shown to be a very successful strategy to fight malignant cancer, especially melanoma [94]. This tumor immune surveillance has mainly been considered in the context of cancer cell destruction. As tumor immune control can, in addition, be explained by non-toxic mechanisms (for an overview, see Figure 3), we focus here on immune-mediated tumor senescence surveillance and explain the underlying events that mediate these non-toxic cellular responses. In this context, we place special emphasis on cytokine-induced senescence (CIS) in tumor cells [95].

The immune system is known to control tissue homeostasis, thereby effectively preventing excessive tissue growth, i.e., tumor development. Normally, cancer development is thought to be prevented through the immune system since malignant cells express distinct markers after their transformation (i.e., tumor antigens) that distinguish them from normal cells, allowing recognition and destruction through immune cells—a concept originally introduced by Burnet and Thomas [96,97]. If this cancer immunosurveillance fails, tumor formation and progression take place via a process known as cancer immunoediting that consists of three stages referred to as: (i) elimination, (ii) equilibrium, and (iii) escape [34,98]. Primarily, the elimination of cancer cells occurs through cytotoxic mechanisms via NKs, CD8^+^ CTLs or neutrophils (see also Section 2). In addition to the direct killing of tumor cells, senescence induction is another (non-toxic) way to prevent tumor growth [99]. Generally, senescence is defined as a state of permanent or at least long-lasting growth arrest that can be induced through a broad variety of stimuli, including DNA damage, oncogenic stress, chemotherapeutic drugs or cytokines [100]. Although senescence is thought to be a strong anti-cancer mechanism, as it initially acts as a barrier that halts the malignant transformation of cells, it may also be accompanied by the formation of a distinct secretome. This SASP contains many pro-inflammatory factors, such as cytokines (e.g., IL-6) and chemokines (e.g., CCL2), that, depending on the context, may (i) reinforce the senescent state of the cells, (ii) induce “bystander” senescence in neighboring cells, (iii) contribute to the immunosurveillance of the senescent cells or (iv) on the other hand, fuel cancer progression [11,101]. During recent years, it has become apparent that cytokines produced and secreted by different immune cells exert a similar function in inducing senescence as components released by senescent cells. The first detailed description of CIS as a new type of immune-mediated tumor control was published almost a decade ago [25]. Braumüller et al. showed in a mouse model of multistage carcinogenesis that the adoptive transfer of tumor-associated antigen (TAA)-specific Th1 cells efficiently stops the growth of pancreatic β-cancer cells without destroying the tumor. While tumor development progressed in mice that received a sham treatment only, the combined action of the cytokines IFN-γ and TNF released by the transferred CD4^+^ Th1 cells led to senescence induction. Aside from our own findings, senescence induction through cytokines has also been reported in the literature by others, as outlined in the next section.

## 4. Senescence Induction in the TME

**Figure 4:**
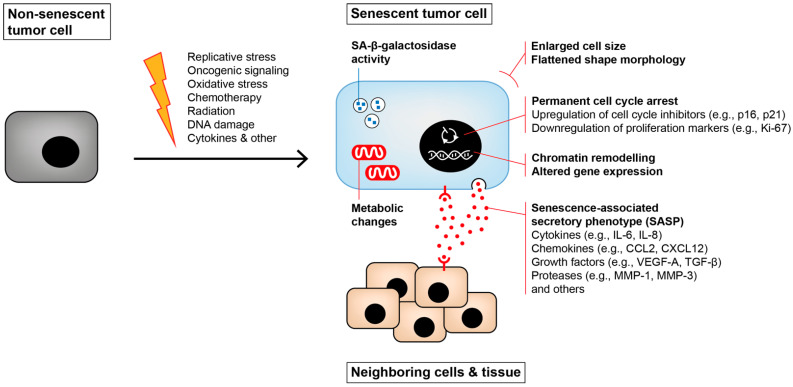
Senescence induction and its impact on neighboring cells. After encountering a senescence trigger (orange lightning), the cells start to change. They adopt a flattened morphology and enlarge in size. Besides being growth-arrested, the cells show increased activity of the senescence-associated β-galactosidase (SA-β-gal), metabolic changes, chromatin remodeling and an altered gene expression, including the formation of a senescence-associated secretory phenotype (SASP). This SASP then acts in an autocrine or paracrine manner, influencing the senescent cells themselves as well as neighboring cells in the tissue.

In addition to other recent work dealing with the role of senescence and its implications for the TME [102,103,104,105], we focus here on the less-described CIS, including the molecular mechanisms and their regulation that mediate this cellular response. In addition to the general concept of senescence explained above, we briefly summarize the most important characteristics of cellular senescence in Figure 4. These characteristics are shared by most types of senescent cells independently of the senescence inducer.

### 4.1. Cytokine-Induced Senescence (CIS)

A quite special form of therapeutic senescence induction is CIS. Almost 10 years ago, CIS was discovered as the result of targeted immunotherapy with TAA-specific and non-cytotoxic Th1 cells in the RIP-Tag2 mouse model [25]. Generally, the mice develop endogenous tumors arising from transformed β-cells of the pancreas, which express the simian virus 40 large T antigen (Tag) under control of the rat insulin promoter (RIP). The adoptive transfer of Tag-Th1 cells into tumor-bearing RIP-Tag2 mice induces senescence in vivo, thereby preventing further tumor progression. This therapeutic response was only achieved in the presence of IFN-γ and TNF. The importance of undisrupted cytokine signaling for CIS was demonstrated by the use of knockout mice lacking either TNR receptor 1 (Tnfr1) or the signal transducer and activator of transcription 1 (Stat1).

The underlying molecular mechanisms were uncovered by ex vivo analyses and further in vitro studies with islets and β-cancer cells isolated from pancreata of tumor-bearing mice. In vivo, the T cell therapy led to the induction of important markers associated with growth arrest and senescence (e.g., upregulation of p16, trimethylation of histone 3 at lysine residue 9 (H3K9me3), and nuclear staining for phosphorylated heterochromatin protein 1 gamma (HP1γ)) that was accompanied by the reduced expression of the proliferation marker Ki-67. Similar results were obtained by the direct use of recombinant cytokines IFN-γ and TNF in vitro. The cytokine treatment was also applied in other cancer entities (murine as well as human), and the induction of senescence was achieved by the combination of both cytokines [25].

In this context, another study revealed a different modulation of senescence and the TME that depends on the administration route used for the adoptive transfer of the Th1 cells [106]. Although the therapeutic effects achieved in tumor-bearing RIP-Tag2 mice after intraperitoneal (i.p.) and intravenous (i.v.) application of the TAA-specific Th1 cells were almost equivalent, senescence induction in the tumor cells was enhanced in the i.v. setting. The anti-tumoral response induced by the transferred CD4^+^ T cells was further accompanied by profound changes in the immune constitution of the TME, as seen by the recruitment of B cells and DCs, while CD8^+^ T cell infiltration was reduced and macrophages were depleted [106].

The importance of Th1 cells and their associated effector cytokines IFN-γ and TNF for in vivo senescence induction was also described in other systems apart from the RIP-Tag2 model. Humanized NSG mice expressing a functional immune system with T cells and NKs were subject to tumor engraftment with the human rhabdomyosarcoma cell line A204 and subsequent immunotherapy with the NHS-IL12 construct [107]. NHS-IL12 is a fusion protein that consists of an antibody targeting the histones of necrotic cells combined with the functional domains of IL-12, a cytokine that is able to mediate IFN-γ-driven immune responses. The therapy was further supported by the administration of engineered IL-2 and IL-7 and efficiently induced anti-tumor immunity in the sarcoma-bearing mice [107]. The resulting tumor remission and long-term survival of the xenografts were not only caused by the immune-mediated senescence induction found in the cancer cells but also attributed to the induction of myogenic differentiation.

A follow-up study presented by the same group revealed that the NHS-IL12 therapy in combination with local tumor irradiation led to improved survival and systemic cancer control [108]. This treatment regimen increases the proportion of necrotic cells due to the irradiation, thereby enhancing intratumoral immunity. Again, both tumor cell senescence and differentiation were observed in the humanized sarcoma-bearing mice as the major consequences of the therapy in vivo. This T cell-driven anti-tumor response was also reproduced in vitro by analyzing different human cancer cell lines. The underlying mechanisms that provoke the measured effects were identified to be dependent on the Th1 cell cytokines IFN-y and TNF. Therefore, CIS can result from novel therapeutic approaches that link, for instance, radiotherapy to an otherwise sole but still efficient immunotherapy [108]. In the context of cell-based immunotherapy, it was also recently shown that gamma delta (γδ) T cells bear a promising anti-tumor activity [109]. The study demonstrated that the TCR-independent stimulation of γδ T cells with cytokines such as IL-2, IL-12, and IL-18 enhanced their anti-tumoral potential. This is achieved as γδ T cells do not only induce apoptosis through cytotoxic factors such as granzymes or perforin, but also tumor cell senescence through the production of IFN-γ and TNF [109].

A study of tumor samples derived from colorectal cancer patients revealed a modification of the immune cell infiltrate in the TME during dissemination and peritoneal carcinomatosis [110]. In contrast to primary tumors, the cells of the metastatic lesions showed a reduced proliferation, enhanced senescence markers, and a different immunological composition. On the one hand, the presence of pro-angiogenic factors like VEGF-A and the increase in B cells and follicular Th cells promotes neovascularization, while on the other hand, the NK cell-mediated immune surveillance of peritoneal carcinomatosis via upregulated levels of IFN-γ and TNF takes place, including the induction of cancer cell senescence [110].

In addition to the functional evidence for immune-mediated senescence induction that was derived from complex in vivo studies or patients, other analyses focused on the mechanistic details in cell culture-based works. Different reports—including our own work—could show that the direct application of cytokines has a similar senescence-inducing effect compared with the action of certain immune cells that release these factors [25,107,111]. Although most reports focus on the effects mediated by IFN-γ and TNF, either alone or in combination, other cytokines have also been associated with the induction of senescence. Interestingly, CIS was not only limited to cancerous cells; it was also described in other cell types. For instance, the senescence of in vitro cultured biliary epithelial cells treated with the pro-inflammatory cytokines IFN-β, IFN-γ or TNF was shown to rely on the activation of the ataxia telangiectasia-mutated (ATM) pathway [112]. Upon induction of oxidative stress via the cytokine or H_2_O_2_ treatment, the ATM kinase was phosphorylated, which in turn triggered the activation of p53 and downstream expression of p21, finally mediating senescence. Although DNA damage was not analyzed in the context of this report, it was already shown by Moiseeva et al. that in contrast to a temporary stimulation with IFN-β, the prolonged exposure of fibroblasts to this cytokine leads to a ROS-triggered DNA damage response and the p53-dependent induction of senescence [113].

The role of type I interferons such as IFN-α and IFN-β in senescence induction was recently reviewed [114]. Since most other studies of CIS are based on the use of IFN-γ and the related interferon signaling response, we now focus on these reports. A study performed by Kim et al. related the induction of senescence in human endothelial cells via prolonged IFN-γ exposure to a p53-mediated DNA damage response [115]. They showed increased staining for the senescence-associated β-galactosidase (SA-β-gal) activity as well as the formation of a G0/G1 arrest for in vitro-treated human umbilical vascular endothelial cells (HUVECs) as a result of oxidative stress and the accumulation of DNA damage. The cell cycle arrest was mediated via upregulated protein levels of p53 and its downstream target p21. In primary human melanocytes, a similar phenotype was described after persistent treatment with IFN-γ [116]. Wang et al. detected intracellular accumulation of ROS within the stimulated melanocytes that was further accompanied by a loss in viability and the induction of apoptotic cell death as well as senescence. The observed cell cycle arrest in G1 was mediated by an increased expression of the p21 protein and essentially required functional interferon signaling via Janus kinase (JAK) 2 and STAT1. The phenotype of senescent melanocytes was further characterized by an altered morphology and pigmentation, enhanced SA-β-gal activity, and the secretion of IL-6 and heat shock protein (HSP)-70 [116]. Hubackova et al. extended the analysis to the effects induced by IFN-γ in different human and murine cell types on the molecular level. Besides the general aspects, such as the formation of oxidative stress and DNA damage, senescence induction via IFN-γ (and in some cases also TNF) was attributed to underlying TGF-β/SMAD signaling [117]. Mechanistically, the cytokine treatment leads to the induction of the NADPH oxidases Nox1 & Nox4 (with the latter being of major importance) via activated JAK/STAT signaling and the secretion of TGF-β that acts in an autocrine and paracrine manner. The simultaneous suppression of adenine nucleotide translocase 2 (ANT2) then further contributes to the accumulation of ROS and genotoxic stress, finally leading to CIS [117].

A previous study of the same group already illustrated the effect of secreted factors in the context of drug-induced senescence [118]. Although cytokines such as IFN-γ or TNF were not used as senescence inducers in the first place, it was shown that the formation of a SASP containing such factors could induce “bystander” senescence in the neighboring cells via paracrine signaling. Such “bystander” effects were also analyzed and compared for senescence that was induced via the chemotherapeutic agent docetaxel (DTX) or cytokines [119]. Using the murine cell lines B16F10 (melanoma) and TC-1 (virus-transformed lung epithelial cells), it was shown that DTX leads to a p21-mediated senescence program in both cell types, which further includes the formation of a SASP capable of inducing “bystander” senescence. Interestingly, the cytokine cocktail of IFN-γ and TNF was not able to permanently arrest the cancer cells. As only the B16F10 cells responded to the initial treatment, e.g., by the upregulation of p21 and other markers, it was also demonstrated that these cells began to grow again after withdrawal of the cytokines. Moreover, the cytokine-treated B16F10 cells formed tumors in vivo and lacked the ability to induce paracrine senescence.

Funck et al. reported senescence induction in human melanoma cells via crosstalk of innate immune cells [120]. Based on the observation that stage I melanoma was characterized by an accumulation of non-classical monocytes (i.e., slanMo), whereas stage III melanoma expressed higher numbers of NKs, their interaction was experimentally analyzed. It was shown that NK migration occurs in response to cell culture supernatants of slanMo containing CXCL8 (also known as IL-8). Co-cultures of both cell types induced the production of IFN-γ and TNF, especially after stimulation with Toll-like receptor (TLR) ligands. The high cytokine level produced in the latter setting was able to induce senescence in various melanoma cell lines that was further accompanied by the expression of a SASP. Therefore, the formation of a TME with senescence-inducing properties via the innate immune defense (represented here by the interaction of slanMo and NKs) is expected by the authors [120]. Besides melanoma, the senescence-inducing properties of IFN-γ and TNF were also demonstrated for other tumors. For instance, a dose-dependent induction of senescence was observed for the combination of these Th1 cell cytokines in different breast cancer cell lines [121]. Moreover, the additional inhibition of human epidermal growth factor receptor 2 (HER2) activity (either by a targeted knockdown or the use of monoclonal antibodies) enhanced the cytokine-mediated response as shown by the induction of tumor cell senescence and apoptosis. In vitro co-culture experiments using patient-derived CD4^+^ T cells primed with HER2 peptides and breast cancer cells with HER2 overexpression confirmed the observed effects. While only a minimal response was induced by IFN-γ and TNF in triple-negative breast cancer cells, combined treatment with an epidermal growth factor receptor (EGFR) inhibitor was able to overcome these limitations. On the molecular level, the cytokines triggered the activation of transcription factor STAT1 through serine and tyrosine phosphorylation, whereas STAT3 activity was reduced [121].

The importance of STAT proteins in the context of CIS was also highlighted by Kandhaya-Pillai et al. [122]. In their study, TNF alone was able to induce senescence in HUVECs, which was characterized by a permanent growth arrest, increased SA-β-gal activity and the expression of p16 and p21. The TNF treatment led further to the production of ROS and lesions with persistent DNA damage. Interestingly, this TNF-mediated senescence program included the induction of a gene expression profile with an interferon signature as well as the activation of an autocrine and STAT-dependent feedback loop that enhanced the secretion of cytokines. This process critically involved the activity of STAT1 and STAT3, both signaling molecules within the JAK/STAT pathway. Experiments using a JAK inhibitor did not prevent the induction of a growth arrest but altered the cellular response to TNF [122]. It has also been found that other immune cells and their cytokines are able to induce senescence, as in the case of Th17 cells and IL-17 [123,124] or even the IL-32 isoform θ that was recently discovered [125]. An overview regarding the different models, cytokines, and induced responses is presented in Table 1.

In addition to the direct effects of the cytokines, immune-mediated cancer control and senescence induction can also be achieved and reinforced by the use of immune checkpoint inhibitors [26]. While blocking antibodies directed against LAG-3 and PD-L1 were sufficient to induce senescence in tumor cells, a combination with an adoptive transfer of TAA-specific Th1 cells further increased this effect. These findings clearly demonstrated that interfering with negative regulators of the immune system either expressed on certain immune cells (i.e., LAG-3) or on tumor cells (i.e., PD-L1) is able to restore an anti-tumor response that induces protective cancer control through the senescence barrier that leads to a stable growth arrest instead of a complete regression. Recent reports further showed the consequences of immune checkpoint blockade (ICB) on other cell types upon treatment in a mouse lymphoma model [126,127]. The antibodies used for ICB had, for instance, favorable effects on immune cells. T cells showed an improved function (e.g., by their cytokine production) and were relevant for longtime survival, whereas NKs also contributed to delayed tumor progression, as their number increased upon ICB treatment, as did their proliferation and production of IFN-γ. Therefore, tumor development in ICB responders is controlled by T cells and NKs that produce effector cytokines leading to tumor cell senescence [126]. Moreover, ICB also exerted an influence on DCs: in response to IFN-γ produced by T cells and NKs, tumor-infiltrating DCs expressed more co-stimulatory molecules and a higher IL-12/IL-10 ratio. Both effects favored T cell-based immunity, as the DCs showed an improved capability of presentation and the secretion of factors that favor a Th1 cell anti-tumor immune response [127]. Although ICB is generally associated with improved therapeutic effects, and many cancer patients could already benefit from its application, there are still variations in the treatment response that rely on several factors, such as alterations of the TME and the immune system, including the occurrence of immuno-senescence, which is subject of the next section [94,128]. In addition, strategies to overcome the limited treatment responses of ICB could include the use of other inhibitors targeting certain kinases, such as the cyclin-dependent kinases (CDK) 4/6, which are also able to induce senescence in cancer cells [129,130,131,132,133].

### 4.2. Senescence Induction in Cells of the Immune System

Senescence induction is not limited to cancerous cells and can also occur in immune cells. Checkpoint inhibitor therapies rely on T cells as the main players against cancer. However, the efficiency of immunotherapies varies dramatically between different tumor entities and tumor sites [134], although in most tumors, large numbers of lymphocytes infiltrate. There are four possible reasons for the variable response rates, tolerance, anergy, exhaustion or senescence. Although plenty of studies deal with tolerance, anergy or exhaustion, very little is known about T cell senescence in the TME [135]. Naïve CD8^+^ T cells have to be activated by DCs that express the surface markers CD70 and CD80/CD86. CD70 and CD80/CD86 bind to the CD27 and CD28 receptors on the surface of the T cell, providing the co-stimulatory signal for effective T cell activation [136]. Senescent T cells, independent of the kind of senescence, downregulate or lose CD27 and CD28 receptors. In several studies, CD8^+^ T cells from older adults had dramatically decreased CD28 expression compared with CD8^+^ T cells from younger adults [137]. At the same time, senescent T cells start to express NK cell-related receptors like natural killer group 2 member D (NKG2D), killer cell immunoglobulin-like receptors (KIRs), CD56, CD57 or CD94. Senescent T cells seem to adopt a state between adaptive immunity and innate immunity that is unique to T cell senescence [138]. In contrast to senescence, exhausted T cells express only inhibitory receptors of the CD28 family of co-stimulatory molecules like PD-1 and CTLA-4 and checkpoint inhibitor molecules like TIM-3 or LAG-3 but no NK cell-related receptors [135]. While anergic and exhausted T cells are metabolically hypoactive, senescent T cells are considered metabolically hyperactive. Senescent T cells produce a SASP with pro-inflammatory cytokines like IFN-γ or TNF.

Senescent T cells display heterogeneous roles in the TME, ranging from immunosuppressive to anti-tumorigenic activities. In breast cancer patients, killer cell lectin-like receptor G1 (KLRG-1)^+^CD57^+^CD4^+^ and CD8^+^ senescent T cells that produce more effector cytokines, granzyme B and perforin accumulate in peripheral blood and in the tumor. The expression of CD4, KLRG-1, and CD57 correlates with increased overall survival for breast cancer patients [139]. Contrary to the beneficial role of KLRG-1^+^CD57^+^CD4^+^ senescent Th cells in breast cancer patients, Ye et al. showed that tumor-derived γδ T cells induce senescence in CD4^+^ T cells and also in DCs that were no longer able to process and present tumor antigens to T cells. In this study, the induction of senescent T cells together with senescent DCs suppressed immune responses against the malignant breast cancer cells [140]. Induction of immunosenescence is not limited to induced Tregs, but it is also found in naturally occurring Tregs [141,142,143]. One important signaling pathway for controlling T cell senescence is the p38 mitogen-activated protein kinase (MAPK) pathway [144,145,146]. As senescent T cells downregulate the co-stimulatory molecules, CD27 and CD28, activation of p38 MAPK cannot be induced by these factors; instead, it must be induced by pro-inflammatory cytokines like IFN-γ and TNF. Immune suppressive activities of senescent T cells are not restricted to breast cancer but can be found in lung cancer, colorectal cancer, ovarian cancer, head and neck cancer, melanoma, endometrial carcinoma, and multiple myeloma [143]. Together, the induction of T cell senescence within the TME is thought to be, in most cases, tumor-promoting by helping cancer cells to escape elimination [146].

## 5. Immunosurveillance of Senescent Cells

The endogenous surveillance through the immune system is not only limited to tumors; it is also capable of detecting and eliminating senescent cells. Senescent cells in tissue repair and produce chemokines that attract immune cells, which eliminate senescent cells and facilitate tissue repair. Senescent cells in wound healing produce chemokines that attract NKs, neutrophils, DCs, monocytes, macrophages, B cells and T cells that efficiently eliminate all senescent cells [147]. Although senescent cancer cells produce and secrete comparable chemokines that should attract the same immune cells as in wound healing, elimination often fails, and senescent cancer cells accumulate within tumors [148,149]. The reasons for the failure of immune cells to clear senescent tumor cells are ill-defined. The group of van Deursen described that only the induction of senescence by p53/p21 leads to a SASP that attracts macrophages to cells with elevated p21. For immunosurveillance, the chemokine CXCL14 was necessary. This chemokine was part of the p21-induced SASP but not of the p16-induced SASP [150]. As several cancer types harbor p53 mutations, and the induction of senescence in these tumor cells can only occur via p16, this could be an explanation for the failure to clear senescent cancer cells. Another explanation for the hindered elimination of senescent cells could also be reflected by the overall process of aging. The risk of developing cancer rises in persons older than 60 years and then declines in persons older than 85 years of age, probably due to a massive reduction of the proliferative potential [128]. Cancer is an age-related disease, and one of the most important factors for this is senescent and dysfunctional immune cells like T cells [151]. There are several molecular hallmarks of T cell aging, including mitochondrial dysfunction, genetic alterations, repertoire reduction, naïve-memory imbalance, lack of plasticity, inflammation or even T cell senescence [152]. Senescent T cells acquire a SASP with pro-inflammatory cytokines like TNF and osteopontin. In tumor immune responses, TILs play an important role. A study by Sceneay et al. demonstrated in a model of triple-negative breast cancer that ICB targeting CTLA-4 and PD-L1 was less efficient in old mice due to immune dysfunction [153]. Therefore, immunoaging is an additional factor that impacts cancer treatment strategies as well as the immunosurveillance of senescent cells in elderly patients. Recently, the therapeutic efficacy of T cells with a chimeric antigen receptor (CAR) targeting a protein on the surface of senescent cells was demonstrated [154]. After contact with the urokinase-type plasminogen activator receptor (uPAR), these CAR T cells efficiently eliminate the senescent cells and thereby ameliorate certain pathological conditions associated with senescence. The use of such engineered immune cells could be a promising strategy to overcome the occurring limitations of senescence immunosurveillance.

## 6. Conclusions and Perspectives

Senescence is considered a tumor-suppressive mechanism that acts as a natural barrier against cancer formation. However, experimental evidence demonstrated that escape mechanisms exist, which provide an exit from the cellular growth arrest. The therapeutic induction of senescence provides yet another opportunity in the adjuvant treatment of cancer. Parts of this anti-cancer concept are the immune-mediated tumor control by CIS, as well as the senescence immunosurveillance that eliminates senescent cells. Since such therapeutic interventions influence the cancer cells and the surrounding microenvironment, including the stoma and cells of the immune system, strategies to selectively remove senescent cells are now extensively studied [155,156,157,158]. In particular, the still-emerging field of senolytic agents that enable a targeted clearance of senescent cells either by the use of compounds such as Bcl-2 inhibitors or even the application of modified immune cells (e.g., senolytic CAR T cells) adds to the feasibility of senescence-inducing therapies. Such therapeutic regimens would first stop cancer progression through the establishment of the senescence barrier, followed by the controlled removal of the senescent cells to prevent deleterious effects of the SASP and the potential risk of relapse (for a detailed overview, see [159]). Therefore, a careful and context-dependent evaluation is needed when harnessing senescence as a matter of choice in the clinical setting of future cancer therapy.

## Figures and Tables

**Table 1:** Overview of model systems describing cytokine-mediated senescence induction.

Cell Type	Inducer(s)	Response (Mechanism of Growth Arrest)	Refs.
Human umbilical vein endothelial cells (HUVECs)	IFN-γ	Induction of senescence (via oxidative stress & DNA damage)	[115]
Murine intrahepatic biliary epithelial cells (mBECs)	IFN-β/IFN-γ/TNF	Induction of senescence (via oxidative stress & ATM/p53 pathway)	[112]
Murine pancreatic β-cell tumors, murine and human cancer cell lines, primary human cancer cells	Th1 cells/IFN-γ + TNF	Induction of senescence (via p16/Rb pathway)	[25]
Human primary melanocytes	IFN-γ	Induction of senescence & apoptosis (via oxidative stress & p21 upregulation)	[116]
Human rhabdomyosarcoma (cell lines and primary cancer cells)	Tumor-targeted IL-12 (via secretion of IFN-γ + TNF)	Induction of senescence & differentiation (via p16 or p21 upregulation)	[107]
Human peritoneal carcinomatosis of colorectal cancer	Immune cell interactions in the TME (via secretion of IFN-γ + TNF)	Induction of senescence (via p21 upregulation)	[110]
Human cancer cell lines (breast and cervix), primary human fibroblasts	IFN-γ	Induction of senescence (via oxidative stress, DNA damage, and TGF-β/SMAD signaling)	[117]
Human umbilical vein endothelial cells (HUVECs)	TNF	Induction of senescence (via oxidative stress, DNA damage, and JAK/STAT signaling)	[122]
Human breast cancer cell lines	Th1 cells/IFN-γ + TNF (also combined with different antibodies)	Induction of senescence & apoptosis (via p15 & p16 upregulation)	[121]
Murine melanoma cell line	IFN-γ + TNF	Induction of reversible senescence (via p21 upregulation)	[119]
Murine B cell lymphoma, murine pancreatic β-cell tumors	Immune checkpoint blockade therapy (also combined with adoptive Th1 cell transfer [26])	Induction of senescence (via p16 or p21 upregulation)	[26,126]
Human cancer cell lines (bladder, melanoma, and breast)	IL-2/IL-12/IL-18-stimulated γδ T cells (via secretion of IFN-γ + TNF)	Induction of senescence (via p21 upregulation)	[109]
Human melanoma cell lines	Co-culture-derived supernatants from non- classical monocytes (slanMo) and NK cells (via secretion of IFN-γ + TNF)	Induction of senescence (via p21 upregulation)	[120]
Murine aortic endothelial cells (MAECs)	Th17 cells/IL-17A	Induction of senescence (via NF-κB/p53/Rb pathway)	[124]
Human breast cancer cell line	IL-32θ	Induction of senescence (mechanism unclear)	[125]

## References

[1] Carter S.B. (1968). Tissue Homeostasis and the Biological Basis of Cancer. Nature.

[2] Campisi J. (2013). Aging, cellular senescence, and cancer. Annu. Rev. Physiol..

[3] López-Otín C., Blasco M.A., Partridge L., Serrano M., Kroemer G. (2013). The hallmarks of aging. Cell.

[4] Muñoz-Espín D., Serrano M. (2014). Cellular senescence: From physiology to pathology. Nat. Rev. Mol. Cell Biol..

[5] Hernandez-Segura A., Nehme J., Demaria M. (2018). Hallmarks of Cellular Senescence. Trends Cell Biol..

[6] Calcinotto A., Kohli J., Zagato E., Pellegrini L., Demaria M., Alimonti A. (2019). Cellular Senescence: Aging, Cancer, and Injury. Physiol. Rev..

[7] Milanovic M., Fan D.N.Y., Belenki D., Däbritz J.H.M., Zhao Z., Yu Y., Dörr J.R., Dimitrova L., Lenze D., Monteiro Barbosa I.A. (2018). Senescence-associated reprogramming promotes cancer stemness. Nature.

[8] Lee S., Schmitt C.A. (2019). The dynamic nature of senescence in cancer. Nat. Cell Biol..

[9] Saleh T., Tyutyunyk-Massey L., Gewirtz D.A. (2019). Tumor Cell Escape from Therapy-Induced Senescence as a Model of Disease Recurrence after Dormancy. Cancer Res..

[10] Saleh T., Bloukh S., Carpenter V.J., Alwohoush E., Bakeer J., Darwish S., Azab B., Gewirtz D.A. (2020). Therapy-Induced Senescence: An “Old” Friend Becomes the Enemy. Cancers.

[11] Coppé J.P., Desprez P.Y., Krtolica A., Campisi J. (2010). The senescence-associated secretory phenotype: The dark side of tumor suppression. Annu. Rev. Pathol..

[12] Tchkonia T., Zhu Y., van Deursen J., Campisi J., Kirkland J.L. (2013). Cellular senescence and the senescent secretory phenotype: Therapeutic opportunities. J. Clin. Investig..

[13] Prata L., Ovsyannikova I.G., Tchkonia T., Kirkland J.L. (2018). Senescent cell clearance by the immune system: Emerging therapeutic opportunities. Semin. Immunol..

[14] Zampetidis C.P., Galanos P., Angelopoulou A., Zhu Y., Polyzou A., Karamitros T., Kotsinas A., Lagopati N., Mourkioti I., Mirzazadeh R. (2021). A recurrent chromosomal inversion suffices for driving escape from oncogene-induced senescence via subTAD reorganization. Mol. Cell.

[15] Zampetidis C.P., Papantonis A., Gorgoulis V.G. (2022). Escape from senescence: Revisiting cancer therapeutic strategies. Mol. Cell. Oncol..

[16] van Deursen J.M. (2014). The role of senescent cells in ageing. Nature.

[17] Xue W., Zender L., Miething C., Dickins R.A., Hernando E., Krizhanovsky V., Cordon-Cardo C., Lowe S.W. (2007). Senescence and tumour clearance is triggered by p53 restoration in murine liver carcinomas. Nature.

[18] Tabin C.J., Bradley S.M., Bargmann C.I., Weinberg R.A., Papageorge A.G., Scolnick E.M., Dhar R., Lowy D.R., Chang E.H. (1982). Mechanism of activation of a human oncogene. Nature.

[19] Fearon E.R., Vogelstein B. (1990). A genetic model for colorectal tumorigenesis. Cell.

[20] Hanahan D., Weinberg R.A. (2000). The hallmarks of cancer. Cell.

[21] Hanahan D., Weinberg R.A. (2011). Hallmarks of cancer: The next generation. Cell.

[22] Pickup M.W., Mouw J.K., Weaver V.M. (2014). The extracellular matrix modulates the hallmarks of cancer. EMBO Rep..

[23] Caon I., Bartolini B., Parnigoni A., Caravà E., Moretto P., Viola M., Karousou E., Vigetti D., Passi A. (2020). Revisiting the hallmarks of cancer: The role of hyaluronan. Semin. Cancer Biol..

[24] Hanahan D. (2022). Hallmarks of Cancer: New Dimensions. Cancer Discov..

[25] Braumüller H., Wieder T., Brenner E., Aßmann S., Hahn M., Alkhaled M., Schilbach K., Essmann F., Kneilling M., Griessinger C. (2013). T-helper-1-cell cytokines drive cancer into senescence. Nature.

[26] Brenner E., Schörg B.F., Ahmetlić F., Wieder T., Hilke F.J., Simon N., Schroeder C., Demidov G., Riedel T., Fehrenbacher B. (2020). Cancer immune control needs senescence induction by interferon-dependent cell cycle regulator pathways in tumours. Nat. Commun..

[27] Ortiz-Montero P., Londoño-Vallejo A., Vernot J.P. (2017). Senescence-associated IL-6 and IL-8 cytokines induce a self- and cross-reinforced senescence/inflammatory milieu strengthening tumorigenic capabilities in the MCF-7 breast cancer cell line. Cell Commun. Signal..

[28] Nejman D., Livyatan I., Fuks G., Gavert N., Zwang Y., Geller L.T., Rotter-Maskowitz A., Weiser R., Mallel G., Gigi E. (2020). The human tumor microbiome is composed of tumor type-specific intracellular bacteria. Science.

[29] Balkwill F.R., Capasso M., Hagemann T. (2012). The tumor microenvironment at a glance. J. Cell Sci..

[30] Mendes B.B., Sousa D.P., Conniot J., Conde J. (2021). Nanomedicine-based strategies to target and modulate the tumor microenvironment. Trends Cancer.

[31] Ayob A.Z., Ramasamy T.S. (2018). Cancer stem cells as key drivers of tumour progression. J. Biomed. Sci..

[32] Reya T., Morrison S.J., Clarke M.F., Weissman I.L. (2001). Stem cells, cancer, and cancer stem cells. Nature.

[33] Fujimaki K., Yao G. (2020). Cell dormancy plasticity: Quiescence deepens into senescence through a dimmer switch. Physiol. Genomics.

[34] Dunn G.P., Bruce A.T., Ikeda H., Old L.J., Schreiber R.D. (2002). Cancer immunoediting: From immunosurveillance to tumor escape. Nat. Immunol..

[35] Priestley P., Baber J., Lolkema M.P., Steeghs N., de Bruijn E., Shale C., Duyvesteyn K., Haidari S., van Hoeck A., Onstenk W. (2019). Pan-cancer whole-genome analyses of metastatic solid tumours. Nature.

[36] Nguyen D.X., Massagué J. (2007). Genetic determinants of cancer metastasis. Nat. Rev. Genet..

[37] Labani-Motlagh A., Ashja-Mahdavi M., Loskog A. (2020). The Tumor Microenvironment: A Milieu Hindering and Obstructing Antitumor Immune Responses. Front. Immunol..

[38] Marcus A., Gowen B.G., Thompson T.W., Iannello A., Ardolino M., Deng W., Wang L., Shifrin N., Raulet D.H. (2014). Recognition of tumors by the innate immune system and natural killer cells. Adv. Immunol..

[39] Judge S.J., Darrow M.A., Thorpe S.W., Gingrich A.A., O’Donnell E.F., Bellini A.R., Sturgill I.R., Vick L.V., Dunai C., Stoffel K.M. (2020). Analysis of tumor-infiltrating NK and T cells highlights IL-15 stimulation and TIGIT blockade as a combination immunotherapy strategy for soft tissue sarcomas. J. Immunother. Cancer.

[40] Russick J., Joubert P.E., Gillard-Bocquet M., Torset C., Meylan M., Petitprez F., Dragon-Durey M.A., Marmier S., Varthaman A., Josseaume N. (2020). Natural killer cells in the human lung tumor microenvironment display immune inhibitory functions. J. Immunother. Cancer.

[41] Judge S.J., Dunai C., Aguilar E.G., Vick S.C., Sturgill I.R., Khuat L.T., Stoffel K.M., Van Dyke J., Longo D.L., Darrow M.A. (2020). Minimal PD-1 expression in mouse and human NK cells under diverse conditions. J. Clin. Investig..

[42] Buckle I., Guillerey C. (2021). Inhibitory Receptors and Immune Checkpoints Regulating Natural Killer Cell Responses to Cancer. Cancers.

[43] Melief C.J. (2008). Cancer immunotherapy by dendritic cells. Immunity.

[44] Martínez-Lostao L., Anel A., Pardo J. (2015). How Do Cytotoxic Lymphocytes Kill Cancer Cells?. Clin. Cancer Res..

[45] St Paul M., Ohashi P.S. (2020). The Roles of CD8(+) T Cell Subsets in Antitumor Immunity. Trends Cell Biol..

[46] Bruni D., Angell H.K., Galon J. (2020). The immune contexture and Immunoscore in cancer prognosis and therapeutic efficacy. Nat. Rev. Cancer.

[47] Balta E., Wabnitz G.H., Samstag Y. (2021). Hijacked Immune Cells in the Tumor Microenvironment: Molecular Mechanisms of Immunosuppression and Cues to Improve T Cell-Based Immunotherapy of Solid Tumors. Int. J. Mol. Sci..

[48] Poncette L., Bluhm J., Blankenstein T. (2021). The role of CD4 T cells in rejection of solid tumors. Curr. Opin. Immunol..

[49] Takeuchi A., Saito T. (2017). CD4 CTL, a Cytotoxic Subset of CD4(+) T Cells, Their Differentiation and Function. Front. Immunol..

[50] Tay R.E., Richardson E.K., Toh H.C. (2021). Revisiting the role of CD4+ T cells in cancer immunotherapy—New insights into old paradigms. Cancer Gene Ther..

[51] Ahrends T., Borst J. (2018). The opposing roles of CD4(+) T cells in anti-tumour immunity. Immunology.

[52] Garner H., de Visser K.E. (2020). Immune crosstalk in cancer progression and metastatic spread: A complex conversation. Nat. Rev. Immunol..

[53] Iglesia M.D., Parker J.S., Hoadley K.A., Serody J.S., Perou C.M., Vincent B.G. (2016). Genomic Analysis of Immune Cell Infiltrates Across 11 Tumor Types. J. Natl. Cancer Inst..

[54] Berntsson J., Eberhard J., Nodin B., Leandersson K., Larsson A.H., Jirström K. (2018). Expression of programmed cell death protein 1 (PD-1) and its ligand PD-L1 in colorectal cancer: Relationship with sidedness and prognosis. Oncoimmunology.

[55] Ishigami E., Sakakibara M., Sakakibara J., Masuda T., Fujimoto H., Hayama S., Nagashima T., Sangai T., Nakagawa A., Nakatani Y. (2019). Coexistence of regulatory B cells and regulatory T cells in tumor-infiltrating lymphocyte aggregates is a prognostic factor in patients with breast cancer. Breast Cancer.

[56] Helmink B.A., Reddy S.M., Gao J., Zhang S., Basar R., Thakur R., Yizhak K., Sade-Feldman M., Blando J., Han G. (2020). B cells and tertiary lymphoid structures promote immunotherapy response. Nature.

[57] Cabrita R., Lauss M., Sanna A., Donia M., Skaarup Larsen M., Mitra S., Johansson I., Phung B., Harbst K., Vallon-Christersson J. (2020). Tertiary lymphoid structures improve immunotherapy and survival in melanoma. Nature.

[58] Fridman W.H., Petitprez F., Meylan M., Chen T.W., Sun C.M., Roumenina L.T., Sautès-Fridman C. (2021). B cells and cancer: To B or not to B?. J. Exp. Med..

[59] Wu K., Lin K., Li X., Yuan X., Xu P., Ni P., Xu D. (2020). Redefining Tumor-Associated Macrophage Subpopulations and Functions in the Tumor Microenvironment. Front. Immunol..

[60] Gardner A., Ruffell B. (2016). Dendritic Cells and Cancer Immunity. Trends Immunol..

[61] Böttcher J.P., Reis e Sousa C. (2018). The Role of Type 1 Conventional Dendritic Cells in Cancer Immunity. Trends Cancer.

[62] Haas L., Obenauf A.C. (2019). Allies or Enemies-The Multifaceted Role of Myeloid Cells in the Tumor Microenvironment. Front. Immunol..

[63] Groth C., Hu X., Weber R., Fleming V., Altevogt P., Utikal J., Umansky V. (2019). Immunosuppression mediated by myeloid-derived suppressor cells (MDSCs) during tumour progression. Br. J. Cancer.

[64] Boettcher S., Manz M.G. (2017). Regulation of Inflammation- and Infection-Driven Hematopoiesis. Trends Immunol..

[65] Fleming V., Hu X., Weber R., Nagibin V., Groth C., Altevogt P., Utikal J., Umansky V. (2018). Targeting Myeloid-Derived Suppressor Cells to Bypass Tumor-Induced Immunosuppression. Front. Immunol..

[66] Jaillon S., Ponzetta A., Di Mitri D., Santoni A., Bonecchi R., Mantovani A. (2020). Neutrophil diversity and plasticity in tumour progression and therapy. Nat. Rev. Cancer.

[67] Coffelt S.B., Kersten K., Doornebal C.W., Weiden J., Vrijland K., Hau C.S., Verstegen N.J.M., Ciampricotti M., Hawinkels L., Jonkers J. (2015). IL-17-producing γδ T cells and neutrophils conspire to promote breast cancer metastasis. Nature.

[68] Shaul M.E., Fridlender Z.G. (2019). Tumour-associated neutrophils in patients with cancer. Nat. Rev. Clin. Oncol..

[69] Raccosta L., Fontana R., Traversari C., Russo V. (2013). Oxysterols recruit tumor-supporting neutrophils within the tumor microenvironment: The many facets of tumor-derived oxysterols. Oncoimmunology.

[70] Reis E.S., Mastellos D.C., Ricklin D., Mantovani A., Lambris J.D. (2018). Complement in cancer: Untangling an intricate relationship. Nat. Rev. Immunol..

[71] Patel S., Fu S., Mastio J., Dominguez G.A., Purohit A., Kossenkov A., Lin C., Alicea-Torres K., Sehgal M., Nefedova Y. (2018). Unique pattern of neutrophil migration and function during tumor progression. Nat. Immunol..

[72] Fridlender Z.G., Sun J., Kim S., Kapoor V., Cheng G., Ling L., Worthen G.S., Albelda S.M. (2009). Polarization of Tumor-Associated Neutrophil Phenotype by TGF-β: “N1” versus “N2” TAN. Cancer Cell.

[73] Gabrilovich D.I., Ostrand-Rosenberg S., Bronte V. (2012). Coordinated regulation of myeloid cells by tumours. Nat. Rev. Immunol..

[74] Coffelt S.B., Wellenstein M.D., de Visser K.E. (2016). Neutrophils in cancer: Neutral no more. Nat. Rev. Cancer.

[75] Eruslanov E.B., Bhojnagarwala P.S., Quatromoni J.G., Stephen T.L., Ranganathan A., Deshpande C., Akimova T., Vachani A., Litzky L., Hancock W.W. (2014). Tumor-associated neutrophils stimulate T cell responses in early-stage human lung cancer. J. Clin. Investig..

[76] Mahiddine K., Blaisdell A., Ma S., Créquer-Grandhomme A., Lowell C.A., Erlebacher A. (2020). Relief of tumor hypoxia unleashes the tumoricidal potential of neutrophils. J. Clin. Investig..

[77] Weis S.M., Cheresh D.A. (2011). Tumor angiogenesis: Molecular pathways and therapeutic targets. Nat. Med..

[78] Stacker S.A., Williams S.P., Karnezis T., Shayan R., Fox S.B., Achen M.G. (2014). Lymphangiogenesis and lymphatic vessel remodelling in cancer. Nat. Rev. Cancer.

[79] Munde P. (2014). Pericytes in Health and Disease. Int. J. Oral Maxillofac. Pathol..

[80] Ribeiro A.L., Okamoto O.K. (2015). Combined Effects of Pericytes in the Tumor Microenvironment. Stem Cells Int..

[81] Sun R., Kong X., Qiu X., Huang C., Wong P.-P. (2021). The Emerging Roles of Pericytes in Modulating Tumor Microenvironment. Front. Cell Dev. Biol..

[82] Kalluri R. (2016). The biology and function of fibroblasts in cancer. Nat. Rev. Cancer.

[83] Liu T., Zhou L., Li D., Andl T., Zhang Y. (2019). Cancer-Associated Fibroblasts Build and Secure the Tumor Microenvironment. Front. Cell Dev. Biol..

[84] Mao X., Xu J., Wang W., Liang C., Hua J., Liu J., Zhang B., Meng Q., Yu X., Shi S. (2021). Crosstalk between cancer-associated fibroblasts and immune cells in the tumor microenvironment: New findings and future perspectives. Mol. Cancer.

[85] Briquez P.S., Hubbell J.A., Martino M.M. (2015). Extracellular Matrix-Inspired Growth Factor Delivery Systems for Skin Wound Healing. Adv. Wound Care.

[86] Piersma B., Hayward M.K., Weaver V.M. (2020). Fibrosis and cancer: A strained relationship. Biochim. Biophys. Acta Rev. Cancer.

[87] Chang J., Chaudhuri O. (2019). Beyond proteases: Basement membrane mechanics and cancer invasion. J. Cell Biol..

[88] Mylonas C.C., Lazaris A.C. (2014). Colorectal cancer and basement membranes: Clinicopathological correlations. Gastroenterol. Res. Pract..

[89] Ghosh D., Mejia Pena C., Quach N., Xuan B., Lee A.H., Dawson M.R. (2020). Senescent mesenchymal stem cells remodel extracellular matrix driving breast cancer cells to a more-invasive phenotype. J. Cell Sci..

[90] Blokland K.E.C., Pouwels S.D., Schuliga M., Knight D.A., Burgess J.K. (2020). Regulation of cellular senescence by extracellular matrix during chronic fibrotic diseases. Clin. Sci..

[91] Levi N., Papismadov N., Solomonov I., Sagi I., Krizhanovsky V. (2020). The ECM path of senescence in aging: Components and modifiers. FEBS J..

[92] Sporn M.B. (1996). The war on cancer. Lancet.

[93] Gatenby R.A. (2009). A change of strategy in the war on cancer. Nature.

[94] Wieder T., Eigentler T., Brenner E., Röcken M. (2018). Immune checkpoint blockade therapy. J. Allergy Clin. Immunol..

[95] Wieder T., Brenner E., Braumüller H., Bischof O., Röcken M. (2017). Cytokine-induced senescence for cancer surveillance. Cancer Metastasis Rev..

[96] Burnet F.M. (1970). The concept of immunological surveillance. Prog. Exp. Tumor. Res..

[97] Thomas L. (1982). On immunosurveillance in human cancer. Yale J. Biol. Med..

[98] Dunn G.P., Old L.J., Schreiber R.D. (2004). The Immunobiology of Cancer Immunosurveillance and Immunoediting. Immunity.

[99] Wieder T., Braumüller H., Brenner E., Zender L., Röcken M. (2013). Changing T-cell enigma: Cancer killing or cancer control?. Cell Cycle.

[100] Gorgoulis V., Adams P.D., Alimonti A., Bennett D.C., Bischof O., Bishop C., Campisi J., Collado M., Evangelou K., Ferbeyre G. (2019). Cellular Senescence: Defining a Path Forward. Cell.

[101] Sagiv A., Krizhanovsky V. (2013). Immunosurveillance of senescent cells: The bright side of the senescence program. Biogerontology.

[102] Ruhland M.K., Alspach E. (2021). Senescence and Immunoregulation in the Tumor Microenvironment. Front. Cell Dev. Biol..

[103] Yasuda T., Baba H., Ishimoto T. (2021). Cellular senescence in the tumor microenvironment and context-specific cancer treatment strategies. FEBS J..

[104] Liu H., Zhao H., Sun Y. (2021). Tumor microenvironment and cellular senescence: Understanding therapeutic resistance and harnessing strategies. Semin. Cancer Biol..

[105] Takasugi M., Yoshida Y., Hara E., Ohtani N. (2022). The role of cellular senescence and SASP in tumor microenvironment. FEBS J..

[106] Griessinger C.M., Schmid A.M., Sonanini D., Schörg B.F., Jarboui M.A., Bukala D., Mucha N., Fehrenbacher B., Steinhilber J., Martella M. (2019). The administration route of tumor-antigen-specific T-helper cells differentially modulates the tumor microenvironment and senescence. Carcinogenesis.

[107] Schilbach K., Alkhaled M., Welker C., Eckert F., Blank G., Ziegler H., Sterk M., Müller F., Sonntag K., Wieder T. (2015). Cancer-targeted IL-12 controls human rhabdomyosarcoma by senescence induction and myogenic differentiation. Oncoimmunology.

[108] Eckert F., Jelas I., Oehme M., Huber S.M., Sonntag K., Welker C., Gillies S.D., Strittmatter W., Zips D., Handgretinger R. (2017). Tumor-targeted IL-12 combined with local irradiation leads to systemic tumor control via abscopal effects in vivo. Oncoimmunology.

[109] Schilbach K., Welker C., Krickeberg N., Kaißer C., Schleicher S., Hashimoto H. (2020). In the Absence of a TCR Signal IL-2/IL-12/18-Stimulated γδ T Cells Demonstrate Potent Anti-Tumoral Function Through Direct Killing and Senescence Induction in Cancer Cells. Cancers.

[110] Seebauer C.T., Brunner S., Glockzin G., Piso P., Ruemmele P., Schlitt H.J., Geissler E.K., Fichtner-Feigl S., Kesselring R. (2016). Peritoneal carcinomatosis of colorectal cancer is characterized by structural and functional reorganization of the tumor microenvironment inducing senescence and proliferation arrest in cancer cells. Oncoimmunology.

[111] Rentschler M., Chen Y., Pahl J., Soria-Martinez L., Braumüller H., Brenner E., Bischof O., Röcken M., Wieder T. (2018). Nuclear Translocation of Argonaute 2 in Cytokine-Induced Senescence. Cell Physiol. Biochem..

[112] Sasaki M., Ikeda H., Sato Y., Nakanuma Y. (2008). Proinflammatory cytokine-induced cellular senescence of biliary epithelial cells is mediated via oxidative stress and activation of ATM pathway: A culture study. Free Radic. Res..

[113] Moiseeva O., Mallette F.A., Mukhopadhyay U.K., Moores A., Ferbeyre G. (2006). DNA damage signaling and p53-dependent senescence after prolonged beta-interferon stimulation. Mol. Biol. Cell.

[114] Frisch S.M., MacFawn I.P. (2020). Type I interferons and related pathways in cell senescence. Aging Cell.

[115] Kim K.S., Kang K.W., Seu Y.B., Baek S.H., Kim J.R. (2009). Interferon-gamma induces cellular senescence through p53-dependent DNA damage signaling in human endothelial cells. Mech. Ageing Dev..

[116] Wang S., Zhou M., Lin F., Liu D., Hong W., Lu L., Zhu Y., Xu A. (2014). Interferon-γ induces senescence in normal human melanocytes. PLoS ONE.

[117] Hubackova S., Kucerova A., Michlits G., Kyjacova L., Reinis M., Korolov O., Bartek J., Hodny Z. (2016). IFNγ induces oxidative stress, DNA damage and tumor cell senescence via TGFβ/SMAD signaling-dependent induction of Nox4 and suppression of ANT2. Oncogene.

[118] Novakova Z., Hubackova S., Kosar M., Janderova-Rossmeislova L., Dobrovolna J., Vasicova P., Vancurova M., Horejsi Z., Hozak P., Bartek J. (2010). Cytokine expression and signaling in drug-induced cellular senescence. Oncogene.

[119] Sapega O., Mikyšková R., Bieblová J., Mrázková B., Hodný Z., Reiniš M. (2018). Distinct phenotypes and ‘bystander’ effects of senescent tumour cells induced by docetaxel or immunomodulatory cytokines. Int J. Oncol..

[120] Funck F., Pahl J., Kyjacova L., Freund L., Oehrl S., Gräbe G., Pezer S., Hassel J.C., Sleeman J., Cerwenka A. (2020). Human innate immune cell crosstalk induces melanoma cell senescence. Oncoimmunology.

[121] Rosemblit C., Datta J., Lowenfeld L., Xu S., Basu A., Kodumudi K., Wiener D., Czerniecki B.J. (2018). Oncodriver inhibition and CD4(+) Th1 cytokines cooperate through Stat1 activation to induce tumor senescence and apoptosis in HER2+ and triple negative breast cancer: Implications for combining immune and targeted therapies. Oncotarget.

[122] Kandhaya-Pillai R., Miro-Mur F., Alijotas-Reig J., Tchkonia T., Kirkland J.L., Schwartz S. (2017). TNFα-senescence initiates a STAT-dependent positive feedback loop, leading to a sustained interferon signature, DNA damage, and cytokine secretion. Aging.

[123] Faust H.J., Zhang H., Han J., Wolf M.T., Jeon O.H., Sadtler K., Peña A.N., Chung L., Maestas D.R., Tam A.J. (2020). IL-17 and immunologically induced senescence regulate response to injury in osteoarthritis. J. Clin. Investig..

[124] Zhang L., Liu M., Liu W., Hu C., Li H., Deng J., Cao Q., Wang Y., Hu W., Li Q. (2021). Th17/IL-17 induces endothelial cell senescence via activation of NF-κB/p53/Rb signaling pathway. Lab. Investig..

[125] Pham T.-H., Park H.-M., Kim J., Hong J.-T., Yoon D.-Y. (2021). Interleukin-32θ Triggers Cellular Senescence and Reduces Sensitivity to Doxorubicin-Mediated Cytotoxicity in MDA-MB-231 Cells. Int. J. Mol. Sci..

[126] Ahmetlic F., Fauser J., Riedel T., Bauer V., Flessner C., Hömberg N., Hennel R., Brenner E., Lauber K., Röcken M. (2021). Therapy of lymphoma by immune checkpoint inhibitors: The role of T cells, NK cells and cytokine-induced tumor senescence. J. Immunother. Cancer.

[127] Scheuerpflug A., Ahmetlić F., Bauer V., Riedel T., Röcken M., Mocikat R. (2021). The role of dendritic cells for therapy of B-cell lymphoma with immune checkpoint inhibitors. Cancer Immunol. Immunother..

[128] Maggiorani D., Beauséjour C. (2021). Senescence and Aging: Does It Impact Cancer Immunotherapies?. Cells.

[129] Wagner V., Gil J. (2020). Senescence as a therapeutically relevant response to CDK4/6 inhibitors. Oncogene.

[130] Schaer D.A., Beckmann R.P., Dempsey J.A., Huber L., Forest A., Amaladas N., Li Y., Wang Y.C., Rasmussen E.R., Chin D. (2018). The CDK4/6 Inhibitor Abemaciclib Induces a T Cell Inflamed Tumor Microenvironment and Enhances the Efficacy of PD-L1 Checkpoint Blockade. Cell Rep..

[131] Lai A.Y., Sorrentino J.A., Dragnev K.H., Weiss J.M., Owonikoko T.K., Rytlewski J.A., Hood J., Yang Z., Malik R.K., Strum J.C. (2020). CDK4/6 inhibition enhances antitumor efficacy of chemotherapy and immune checkpoint inhibitor combinations in preclinical models and enhances T-cell activation in patients with SCLC receiving chemotherapy. J. Immunother. Cancer.

[132] Ruscetti M., Morris J.P.T., Mezzadra R., Russell J., Leibold J., Romesser P.B., Simon J., Kulick A., Ho Y.J., Fennell M. (2020). Senescence-Induced Vascular Remodeling Creates Therapeutic Vulnerabilities in Pancreas Cancer. Cell.

[133] Willobee B.A., Gaidarski A.A., Dosch A.R., Castellanos J.A., Dai X., Mehra S., Messaggio F., Srinivasan S., VanSaun M.N., Nagathihalli N.S. (2021). Combined Blockade of MEK and CDK4/6 Pathways Induces Senescence to Improve Survival in Pancreatic Ductal Adenocarcinoma. Mol. Cancer Ther..

[134] Moreira A., Gross S., Kirchberger M.C., Erdmann M., Schuler G., Heinzerling L. (2019). Senescence markers: Predictive for response to checkpoint inhibitors. Int J. Cancer.

[135] Huff W.X., Kwon J.H., Henriquez M., Fetcko K., Dey M. (2019). The Evolving Role of CD8(+)CD28(-) Immunosenescent T Cells in Cancer Immunology. Int J. Mol. Sci..

[136] Esensten J.H., Helou Y.A., Chopra G., Weiss A., Bluestone J.A. (2016). CD28 Costimulation: From Mechanism to Therapy. Immunity.

[137] Rodriguez I.J., Lalinde Ruiz N., Llano León M., Martínez Enríquez L., Montilla Velásquez M.D.P., Ortiz Aguirre J.P., Rodríguez Bohórquez O.M., Velandia Vargas E.A., Hernández E.D., Parra López C.A. (2021). Immunosenescence Study of T Cells: A Systematic Review. Front. Immunol..

[138] Seyda M., Elkhal A., Quante M., Falk C.S., Tullius S.G. (2016). T Cells Going Innate. Trends Immunol..

[139] Ramello M.C., Núñez N.G., Tosello Boari J., Bossio S.N., Canale F.P., Abrate C., Ponce N., Del Castillo A., Ledesma M., Viel S. (2021). Polyfunctional KLRG-1(+)CD57(+) Senescent CD4(+) T Cells Infiltrate Tumors and Are Expanded in Peripheral Blood From Breast Cancer Patients. Front. Immunol..

[140] Ye J., Ma C., Hsueh E.C., Eickhoff C.S., Zhang Y., Varvares M.A., Hoft D.F., Peng G. (2013). Tumor-derived γδ regulatory T cells suppress innate and adaptive immunity through the induction of immunosenescence. J. Immunol..

[141] Ye J., Huang X., Hsueh E.C., Zhang Q., Ma C., Zhang Y., Varvares M.A., Hoft D.F., Peng G. (2012). Human regulatory T cells induce T-lymphocyte senescence. Blood.

[142] Liu X., Mo W., Ye J., Li L., Zhang Y., Hsueh E.C., Hoft D.F., Peng G. (2018). Regulatory T cells trigger effector T cell DNA damage and senescence caused by metabolic competition. Nat. Commun..

[143] Liu X., Hoft D.F., Peng G. (2020). Senescent T cells within suppressive tumor microenvironments: Emerging target for tumor immunotherapy. J. Clin. Investig..

[144] Callender L.A., Carroll E.C., Beal R.W.J., Chambers E.S., Nourshargh S., Akbar A.N., Henson S.M. (2018). Human CD8(+) EMRA T cells display a senescence-associated secretory phenotype regulated by p38 MAPK. Aging Cell.

[145] Shi L., Zhao Y., Fei C., Guo J., Jia Y., Wu D., Wu L., Chang C. (2019). Cellular senescence induced by S100A9 in mesenchymal stromal cells through NLRP3 inflammasome activation. Aging.

[146] Zhao Y., Shao Q., Peng G. (2020). Exhaustion and senescence: Two crucial dysfunctional states of T cells in the tumor microenvironment. Cell Mol. Immunol..

[147] Krizhanovsky V., Yon M., Dickins R.A., Hearn S., Simon J., Miething C., Yee H., Zender L., Lowe S.W. (2008). Senescence of activated stellate cells limits liver fibrosis. Cell.

[148] Hoenicke L., Zender L. (2012). Immune surveillance of senescent cells--biological significance in cancer- and non-cancer pathologies. Carcinogenesis.

[149] Lujambio A. (2016). To clear, or not to clear (senescent cells)? That is the question. Bioessays.

[150] Sturmlechner I., Zhang C., Sine C.C., van Deursen E.J., Jeganathan K.B., Hamada N., Grasic J., Friedman D., Stutchman J.T., Can I. (2021). p21 produces a bioactive secretome that places stressed cells under immunosurveillance. Science.

[151] Childs B.G., Durik M., Baker D.J., van Deursen J.M. (2015). Cellular senescence in aging and age-related disease: From mechanisms to therapy. Nat. Med..

[152] Mittelbrunn M., Kroemer G. (2021). Hallmarks of T cell agi.ing. Nat. Immunol..

[153] Sceneay J., Goreczny G.J., Wilson K., Morrow S., DeCristo M.J., Ubellacker J.M., Qin Y., Laszewski T., Stover D.G., Barrera V. (2019). Interferon Signaling Is Diminished with Age and Is Associated with Immune Checkpoint Blockade Efficacy in Triple-Negative Breast Cancer. Cancer Discov..

[154] Amor C., Feucht J., Leibold J., Ho Y.J., Zhu C., Alonso-Curbelo D., Mansilla-Soto J., Boyer J.A., Li X., Giavridis T. (2020). Senolytic CAR T cells reverse senescence-associated pathologies. Nature.

[155] van Deursen J.M. (2019). Senolytic therapies for healthy longevity. Science.

[156] Kirkland J.L., Tchkonia T. (2020). Senolytic drugs: From discovery to translation. J. Intern. Med..

[157] Robbins P.D., Jurk D., Khosla S., Kirkland J.L., LeBrasseur N.K., Miller J.D., Passos J.F., Pignolo R.J., Tchkonia T., Niedernhofer L.J. (2021). Senolytic Drugs: Reducing Senescent Cell Viability to Extend Health Span. Annu. Rev. Pharmacol. Toxicol..

[158] Gasek N.S., Kuchel G.A., Kirkland J.L., Xu M. (2021). Strategies for targeting senescent cells in human disease. Nat. Aging.

[159] Sieben C.J., Sturmlechner I., van de Sluis B., van Deursen J.M. (2018). Two-Step Senescence-Focused Cancer Therapies. Trends Cell Biol..

